# Artificial Intelligence in Healthcare: Perception and Reality

**DOI:** 10.7759/cureus.45594

**Published:** 2023-09-20

**Authors:** Abidemi O Akinrinmade, Temitayo M Adebile, Chioma Ezuma-Ebong, Kafayat Bolaji, Afomachukwu Ajufo, Aisha O Adigun, Majed Mohammad, Juliet C Dike, Okelue E Okobi

**Affiliations:** 1 Medicine and Surgery, Benjamin S. Carson School of Medicine, Babcock University, Ilishan-Remo, NGA; 2 Public Health, Georgia Southern University, Statesboro, USA; 3 Nephrology, Boston Medical Center, Malden, USA; 4 Internal Medicine, Angelic Care Hospital, Abuja, NGA; 5 Ophthalmology, Aminu Kano Teaching Hospital, Kano, NGA; 6 Internal Medicine, All Saints University, Roseau, DMA; 7 Infectious Diseases, University of Louisville, Louisville, USA; 8 Geriatrics, Mount Carmel Grove City Hospital, Grove City, USA; 9 Internal Medicine, University of Calabar, Calabar, NGA; 10 Family Medicine, Larkin Community Hospital Palm Springs Campus, Miami, USA; 11 Family Medicine, Medficient Health Systems, Laurel, USA; 12 Family Medicine, Lakeside Medical Center, Belle Glade, USA

**Keywords:** precision medicine, ai in medicine, deep learning, machine learning, artificial intelligence in healthcare

## Abstract

Artificial intelligence (AI) has birthed the new "big thing" in modern medicine. It promises to bring about safer and improved care that will be beneficial to patients and become a helpful tool in the hands of a skilled physician. Despite its anticipation, however, the implementation and usage of AI are still in their elementary phases, particularly due to legal and ethical considerations that border on "data." These challenges should not be brushed aside but rather be recognized and resolved to enable acceptance by all relevant stakeholders without prejudice. Once these challenges can be overcome, AI will truly revolutionize the field of medicine with improved diagnostic accuracy, a reduction in physician burnout, and an enhanced treatment modality. It is therefore paramount that AI be embraced by physicians and integrated into medical education in order to be well-prepared for our role in the future of medicine.

## Introduction and background

The field of artificial intelligence (AI) is vast, rapidly expanding, and breaking new terrains. AI is narrowing the gap between computers and humans. It "refers to systems that display intelligent behavior by analyzing their environment and acting, with some degree of autonomy, to achieve specific goals" [1]. It accomplishes this by collecting a large amount of data, processing it, and learning from the past to streamline and improve the future. The field of AI was formally established in the 1950s during the Dartmouth Summer Research Project on Artificial Intelligence [2]. However, scientists such as Alan Turing already had AI assimilated into their minds even before it was presented at the Dartmouth Summer Research Project by John McCarthy and Marvin Minsky in 1956. Alan Turing introduced the idea of simulating intelligent behavior and critical thinking using computers in 1950 [3]. In his book "Computers and Intelligence," he proposed a test called the Turing test to assess whether computers could exhibit human-like intelligence. Between the 1950s and 1970s, AI progressed from the development of machines capable of human-like decision-making to the creation of Eliza, an AI that utilized natural language processing, and Shakey, the first electronic person [3]. Then, a period of reduced funding for and interest in AI was observed between the 1970s and the 2000s. Nonetheless, during this period, AI found applications in medicine, such as the causal-associational network (CASNET) model, a consultation program for glaucoma that provided personalized advice to physicians based on patient information [3]. Since its inception, the field has undergone significant improvements in robotics, computer vision, speech recognition, image recognition, and natural language processing.

AI has been adopted in multiple fields for prediction and decision-making. It encompasses a spectrum of learning that includes machine learning, representation learning, deep learning, natural language processing, and computer vision [3]. Machine learning utilizes computer algorithms to recognize patterns from raw data and acquire knowledge independent of human input [4]. The acquired knowledge is used for problem-solving and decision-making in unfamiliar conditions. Machine learning has evolved into deep learning, which is made up of algorithms that form an artificial neural network that can learn and make decisions on its own, similar to the human brain [3]. Deep learning can uncover correlations that are too complex to uncover with machine learning [5]. Currently, deep learning seems to be at the center of a new revolution in unraveling "big" data. Artificial neural networks are AI tools that operate by imitating the human brain by connecting and recognizing complex relationships and patterns between data [6]. A convolutional neural network is a class of artificial neural network that can process data in visualized imagery [6]. Natural language processing applications include speech recognition, text analysis, translation, and other language-related aims [7].

AI is pioneering a new paradigm for healthcare advancement and research. Recent advances within AI, such as increased access to large and complex health data, faster computer processing speed, an increase in the AI talent pool, and the availability of AI tools [5], are making AI an integral part of modern healthcare. While the healthcare industry has appeared to be a slow adopter of the technology, the pace of its implementation is growing exponentially. As an ecosystem, the healthcare industry has realized the importance of adopting AI systems to overcome the ever-increasing shortage of healthcare practitioners, mounting expectations of patients, and technological advancements; improve quality; and achieve optimal health outcomes. There is a consensus that AI has the potential to revolutionize patient care through precision medicine and patient-centered care delivery.

This literature review aims to demonstrate how AI is changing healthcare as we know it by drawing attention to the current and potential use of AI in healthcare. This review also addresses the ethical challenges, common misconceptions, and perceptions of healthcare practitioners and patients regarding AI in healthcare.

## Review

Up to date on artificial intelligence

The most prevalent application of classical machine learning in healthcare is precision medicine, which predicts the treatment protocols that are likely to be successful on a patient based on numerous patient traits and the treatment context [7]. Other current applications of machine learning in healthcare are the e-triage system, a machine learning emergency department triage system, and the targeted real-time early warning (TREW) score for predicting septic shock hours before it occurs [8]. Machine learning and natural learning processing models were used by some researchers to identify cases of community-acquired pneumonia and to establish relationships between free-text notes by clinicians and the pneumonia outcome [8].

Another current application of machine learning in healthcare is virtual nursing; recognizing possibly malignant tumors in radiological images is a common application of deep learning in medicine. As a form of natural learning processing, deep learning is now being employed for speech recognition [7]. Natural learning processing systems can evaluate unstructured clinical notes on patients, create reports, transcribe patient conversations, and perform conversational AI. The examples of experimental and current applications of AI in healthcare are shown below.

Imaging

The introduction of deep neural networks has revolutionized the analysis of medical and histopathologic images. Several studies [9-12] used machine learning and deep learning to interpret medical images from breast cancer recognition to colon cancer detection and neurodegenerative disease imaging. In fact, computational models based on deep neural networks detected cancer in mammograms with comparable accuracy to trained radiologists in some studies [9-11]. Akselrod-Ballin et al. (2019) developed an AI system that combined both machine learning and deep learning. The authors trained this system using 9,611 mammograms and health records of females. The goal was to use this system to predict biopsy malignancy and to differentiate normal from abnormal screening examinations. The researchers integrated both imaging and clinical information into their model. This machine learning-deep learning algorithm yielded an area under the receiver operating characteristic curve (AUC) of 0.91 (95% CI: 0.89, 0.93), with a specificity of 77.3% (95% CI: 69.2, 85.4) at a sensitivity of 87% for malignancy prediction. The AUC for the identification of normal examinations was 0.85 (95% CI: 0.84, 0.86). The results obtained in this study were similar to radiologists' ranges as described by the Breast Cancer Surveillance Consortium benchmark (sensitivity, 75%; specificity, 88%-95%) [11].

Similarly, in a study conducted in Singapore, Ting et al. (2017) trained a deep learning system for the detection of diabetic retinopathy using 76,370 images, glaucoma using 125,189 images, and age-related macular degeneration using 72,610 images. This algorithm was found to have high sensitivity and specificity for identifying diabetic retinopathy, glaucoma, and age-related macular degeneration among retinal images of patients with diabetes who participated in the Singapore National Diabetic Retinopathy Screening Program (SIDRP) between 2010 and 2013. The performance of this deep learning system was on par with the experts' assessment of retinal images [10].

In contrast, the AI system outperformed some experts in a study by Rodriguez-Ruiz et al. (2019). These researchers compared, at a case level, the cancer detection performance of a commercially available AI system to that of 101 radiologists. This AI system was a deep learning convoluted neural network system with classifiers and image analysis algorithms that could detect calcifications and soft tissue lesions. The AI system was trained, validated, and tested using a database containing over 9,000 mammograms with cancer. The authors obtained several digital mammography examinations read by multiple radiologists in retrospective multi-reader multi-case (MRMC) observer studies. The researchers found out that the AUC of the AI system (0.840; 95% CI: 0.820, 0.860) was statistically non-inferior to that of the 101 radiologists (0.814; 95% CI: 0.787, 0.841). The AI system also had higher sensitivity than 55 out of 95 radiologists (57.9%), although its performance fell short of that of the best radiologist [12]. The authors concluded however that the system's overall performance was similar to human performance. Likewise, an artificial neural network was developed by Geddes et al. (1998) to predict the outcome of patients with IgA nephropathy with poor prognosis. The AI system was trained using retrospective data from 54 patients. Six nephrologists from three different centers were also shown the same data set and asked to predict the outcome of IgA nephropathy using the same criteria. The researchers discovered that the system predicted outcomes in patients with IgA nephropathy more accurately than trained nephrologists. The study found that the AI system had an impressive outcome performance, accurately predicting the correct outcome in 87% of cases. It demonstrated a sensitivity of 86.4% and a specificity of 87.5%. In contrast, the trained nephrologists had a mean number of correct predictions of 69.4%, a mean sensitivity of 72%, and a mean specificity of 66% [13].

Precision Medicine

Several strides have been made in the utilization of AI for precision medicine [14-16]. Hermsen et al. (2019) developed and trained a convoluted neural network for the multi-class segmentation of renal tissue in routinely periodic acid-Schiff (PAS)-stained sections. This system picked 92% of all glomeruli in 1,819 nephrectomy samples with 10.4% false positives. Regardless of scanning resolution, fixation, and staining protocols, this AI maintained high segmentation performance and showed potential usage for healthy and diseased tissues providing opportunities for deep learning application in routine diagnostics [16]. Also, Attia et al. (2019) trained a convoluted neural network to identify patients with ventricular dysfunction. The AI was trained using both paired 12-lead ECG and echocardiogram data [17]. When tested on a population of over 52,000 patients, the AI yielded an accuracy of 85.7%, a sensitivity of 86.3%, and a specificity of 85.7%. The researchers are optimistic that the application of AI to ECG may serve as a powerful screening tool for asymptomatic left ventricular dysfunction [17]. Likewise, in a study by Isin and Ozdalili (2017), a deep learning-based system was trained to automatically diagnose ECG arrhythmia by classifying ECGs into normal, paced, or right bundle branch block. The authors found that the system they created had a recognition rate of 98.51% and a testing accuracy of 92% [18].

In a separate study, Rotondano et al. (2011) developed an artificial neural network (Training with Input Selection and Testing {TWIST}) system to predict mortality in patients with nonvariceal upper GI bleeding and compared its predictive performance to the Rockall score [14]. The authors learned that the predictive performance of the AI in mortality prediction was significantly superior to the Rockall score with an area under the curve of 0.95 (0.92-0.98) versus 0.67 (0.65-0.69) (P<0.001). The artificial neural network-based model also had a higher accuracy (96.8% versus 52.9%), sensitivity (83.8% versus 71.4%), and specificity (97.5% versus 52.0%) compared to the complete Rockall score [14].

AI was used to predict the need for additional surgery after the endoscopic resection of T1 colorectal cancer by Ichimasa et al. (2018) by analyzing 45 clinicopathological factors in 690 patients with T1 colorectal cancer [15]. This AI was used to forecast the presence or absence of lymph node metastasis (LNM). The authors uncovered that while sensitivity to the presence of LNM was 100% in all models, compared to conventional guidelines, the specificity of the AI (66%) to the existence of lymph node metastasis was greater than all traditional (American {44%}, European {0%}, and Japanese {0%}) guidelines. The authors also realized that the AI algorithm had a much lower rate (77%) of unnecessary additional surgery attributable to misdiagnosing patients without LNM as LNM-positives compared to the 85%, 91%, and 91% rates found in the American, European, and Japanese guidelines, respectively [15].

Health Informatics

Robotic process automation performs administrative procedures by performing structured digital tasks. They are used in healthcare for repetitive operations such as prior authorization, updated patient data, and billing [7]. An AI-enabled system based on natural language processing and neural networks called Savana was designed by Hernández Medrano et al. (2017) for expanding medical terminologies automatically. The automated and precise digital extraction should aid in the generation of a real-time information engine [19].

Perception toward artificial intelligence

As the conversation around AI continues to grow, there has been a mix of varying reactions among different healthcare stakeholders and the population that they cater to. The rate of ongoing conversation around AI has been found to fluctuate, with an observed increase in social media discussions when there are breakthroughs on AI, reports by the government or affiliated authorities on AI, and entertainment news surrounding AI [20].

While some are thrilled by such news of technology breakthroughs in a field that has been judged over the decades as short-staffed especially among developing countries, many others are of the opinion that AI in itself remains a myth. A study conducted in France among 40 health practitioners and related allies described the enthusiasm shared by health industries and researchers concerning the development of AI. This enthusiasm contrasts that of clinical physicians who were of the opinion that AI is still in its early stage with a yet demonstrable evidence of a well-balanced clinical utilization despite the already existing representation of AI's popular culture, which they attributed to media buzz. The majority shared that AI is being viewed by many as the perfect human solution provider as opposed to its reality as different technological tool sets [21].

Of note, consumers' (patients) preference for the use of automated machines in obtaining medical diagnosis has been an interesting area of research. A clinical hypothesis on the reluctance of AI use by consumers in comparison to obtaining medical care from human medical providers proved true in multiple test scenarios [22]. Consumers were assessed on healthcare utilization, healthcare pricing, and variation in performance by providers. Consumers preferred the use of human providers across the different test levels, even when the automated provider was described as having a superior performance when compared to a human provider [22]. Although one may ponder whether resistance to AI is an absolute or relative fear of the unknown, studies have shown that both patients and physicians largely understand and appreciate the benefits that AI may potentially bring to revolutionizing healthcare. However, most of their opinions share a common ground for adjunct use to human providers as opposed to sole functional AI providers. One such study was a qualitative study with primary care stakeholders (physicians and patients) across Canada using a deliberative dialogue in evaluating how stakeholders want AI to be applied in primary healthcare, the impact on stakeholders' experience, and barriers and concerns [23]. The participants in this study opined that the application of AI to documentation, practice operations, and triage tasks should be adopted while human-centered relations, access, and delivery should be maintained [23]. In another study that assesses the use of AI chatbots in the promotion of behavioral health changes, the possibility for its potential use by the participants in the near future was accepted by less than 50% of the study population. This low acceptance for future use was recorded even in the presence of the improved efficacy of chatbots in helping the participants promote a better lifestyle and the provision of an avenue for safer space expression that chatbots provided with regard to sensitive and personal questions such as sex-related issues and drug use [24].

Despite the knowledge of AI's efficiency and capability in medical diagnosis, a survey by the Pew Research Center showed that six in 10 American adults would be uncomfortable if their physician was to rely on AI, and the effect on patient-physician interpersonal connection would be made worse if AI was used for patients' diagnosis and treatment recommendations [25]. A separate research in China however contrasts that of the American and Canadian population, as study data collated through an application programming interface showed that 59.4% of users opined a positive view of AI, 34.4% expressed a neutral stand, and only 6.2% of users expressed a negative attitude toward the idea of AI [20]. Some of the reasons given by users expressing a positive attitude were the technical advantages of AI in healthcare, hopes of industrial development, and help that AI will provide to human physicians. In contrast, those who expressed negative views of AI attributed their opinions to the immaturity of AI, the lack of trust in AI companies, fear, and privacy issues [20]. Beyond users' perspectives, healthcare experts from a study have shared opinions on the need for more education as regards to AI and with questions on its real-life application in clinical medicine. Many of these experts are also of the belief that the difficulties encountered in other sectors that AI has been applied to may equally be transmissible to the healthcare system and may potentially cause disparity in healthcare [26].

Why we should adopt AI in the 21st century

Although the full potential of AI is continuously undergoing exploration, studies already project that the advent of AI will revolutionize the field of medicine in many ways including improved surgical safety and diagnostic accuracy, reduction in healthcare expenses, and an overall improvement in patient care [27,28].

The magnitude of such benefits and impact may be the reason that necessitates the adoption of AI as an added adjunct in improving global healthcare. Several studies have shown the beneficial impact of the adoption of AI. Beyond the generally negative perception, there is an immense need for more education as regards AI and questions on its real-life application in clinical medicine. Some compelling reasons why AI should be integrated into healthcare are shown below.

Automation and Efficiency

The benefits of AI are not only limited to patients alone but also useful in reducing physician burnout. One of AI's strong suits is its ability to automate repetitive and mundane tasks, thereby providing opportunities to better channel human resources in a much-needed direction. This leads to a reduction in the cost of healthcare and increased human physician efficiency and productivity. AI could potentially help in off-loading certain administrative works that serve as an added pile for healthcare workers, sift through document searches at a faster pace, and function as an automated medical scribe [29].

Enhanced Decision-Making

AI is able to extract medical insights and provide data-driven recommendations through the identification of patterns, trends, and correlations that helps to improve fast and enhanced decision-making toward patient healthcare. For example, the predictive capability of AI may be leveraged in the near future in helping to provide anticipatory decision-making and targeted treatment to high-risk patients as demonstrated by a higher machine prediction risk in predicting the readmission rates of patients who have previously been hospitalized for heart failure [30].

Healthcare Advancement

Machine learning algorithms can help in several aspects of radiological analysis and the early detection and diagnosis of disease pathology. Radiological imaging analyses between human experts and AI have been compared, with an established superior AI outcome in the diagnosis of pneumonia using a convolutional neural trained network, an accurate classification of skin lesions, and the detection of metastatic breast cancer through lymph nodes [28].

Improved User Experience and Personalization

There is a direct relationship between providing personalized patient service and AI as evidenced by the digitization of provider-patient interactions in reducing patient wait time and enabling their comfort. Also, many patients have more personalized access to quick health information through various technology-driven chatbots [24]. AI systems can be developed to predict patient flow, thus allowing healthcare providers to optimize resource allocation and staff management to reduce wait time. Traditionally, scheduling appointments in healthcare facilities has been complex and time-consuming. AI can leverage machine learning algorithms to enhance the scheduling process.

Provision of Up-to-Date Guidelines and Developments

Medical guidelines are constantly evolving and re-modified with new advances and trends in the management of various clinical conditions coming out every day; it may be difficult for physicians to constantly keep up with these new guidelines. AI software therefore becomes a useful tool to keep up with these guidelines [24].

Innovations and Economic Growth

AI possesses the ability to speed up growth in the economy. It has proven to help businesses thrive from the ground level. And for those already in the market, AI has improved competitiveness while also creating market opportunities for a lot of entrepreneurs to analyze and delve into [24].

With a well-mapped-out and synchronized implementation, the adoption of AI has the potential to significantly impact 21st-century healthcare and foster progressive advancement in the field of medicine.

Misconceptions and challenges surrounding AI

A major contributor to the misconceptions surrounding AI may perhaps be attributed to its online sensationalism and/or marketing strategy. It is not uncommon to see headlines that particularly talk in awe of AI as though it was created to replace humans. One of such was a major headline that read, "Are you scared yet, human?" [31]. Little wonder that a survey carried out on the most popular and disturbing myths of AI included references to AI as the incoming superintelligence [32]. Some sensationalism creates unrealistic expectations and perpetuates the notion that AI is all-powerful and potentially dangerous.

The concept of AI in itself was designed to act like or better in efficiency than its human counterparts. Hence, the question that prevails is, will AI replace physicians? Gao et al. (2020) analyzed 200 social media posts on AI replacing human doctors. Eighty percent of the posts supported the narrative that AI could either entirely or partially replace human doctors, and 20% of the posts did not think so [20]. The accuracy, stability, and efficiency of AI were cited as the reasons for AI potentially replacing humans, while the need for empathy, ethics, and the immaturity of AI technology were considered reasons why AI cannot take the place of physicians [20].

Despite all the sensationalism, however, we believe that AI will function only as a helpful guide to human healthcare providers, as with many innovative human inventions. AI is not able to implement an entire job by itself and is, rather, a task-specific tool that will serve to boost but not replace physicians [33]. Furthermore, even though AI has the potential for improved diagnostic accuracy, the obtainable results will still require clinical care management by a human professional [34]. It is therefore paramount to embrace and view AI as having the potential to make healthcare more effective, rather than replacing human physicians.

Despite the need to dispel misconceptions surrounding AI, it would be disingenuous to state that the invention is without its own challenges. Truly, the adoption of AI in healthcare is surrounded by a colossally abundant number of ethical and legal issues. Some of these issues are data protection, data privacy, the risk to confidentiality, liability, blurring the line between physicians' and machines' roles in patient care, accountability of errors, and informed consent to use AI systems. Autonomy, beneficence, nonmaleficence, and justice constitute the foundation of medical ethics. Any of these principles can potentially be violated during the implementation of AI. AI has the potential to infringe upon patient autonomy if decisions are solely determined by algorithms without considering patient preferences.

Since AI algorithms are created from collected data, the issue of data privacy and transparency of data use is fast becoming significant. A recent study by Rahimi et al. (2021) shed light on hindrances faced when using AI in community-based primary healthcare [35]. The authors found that the variability of patient data was identified as a significant obstacle because it may require more work to propose AI systems that can analyze and interpret this diverse data effectively. The lack of uniformity in how health information is recorded and poor interprofessional communication were other factors seen in the study by Rahimi et al. [35]. These factors may affect the integrity of the data fed into the AI model. Similarly, data availability poses a significant barrier as it may hamper AI systems' training, testing, and validation. Without enough data, AI models may lack robustness and exhibit lower accuracy in their predictions.

Also, AI can be subject to algorithmic bias, especially when the data used for training the system does not truly represent the target population [7,36]. This bias can manifest in various aspects, such as gender, race, socio-economic status, or any other characteristics that may be present in the data. Biases in the data collection process, such as biased human judgments or historical prejudices, can also contribute to algorithmic bias. The generalizability of prediction models in terms of their diagnostic accuracy may vary across different groups of populations [37]. However, this sentiment is not shared by some stakeholders. The Pew Research Center survey conducted in 2022 on US adults revealed mixed perceptions of AI's influence on bias. Fifty-one percent of the respondents who admitted that bias based on race or ethnicity is a problem in health and medicine claimed that relying more on AI would improve the issue, and 15% claimed the situation might worsen. Three percent of the respondents did not think that AI would make a difference [25].

Due to an already existing healthcare disparity issue, there have been concerns that discriminatory bias in AI, particularly among minority groups, may challenge its utilization in healthcare. Concerns for equality in healthcare among minority groups were particularly proved true in the case of an algorithm machine that can diagnose both malignant and benign skin moles as efficiently as when compared to qualified, certified dermatologists would have done but with the same lesser diagnostic accuracy among people of color [38].

Because available data sets are the modus operandi in the development of automated learning, populations with lesser access to healthcare may invariably have limited data sets for training purposes, thereby reproducing a systemic healthcare bias for these populations. Another example is seen in the study of the Framingham risk factors, which had overestimations and underestimations in predicting cardiovascular events among the non-white study group [39].

Algorithmic, discriminatory, and systemic biases can impinge on the principle of beneficence and erode public trust in AI systems, creating skepticism and further hindering the adoption of these technologies.

In the case of COVID-19, various data sets encompassing lung images of infected individuals were collected to enable the development of new model algorithms that can potentially improve early diagnosis and severity indication [40]. The utilization of such a data set is, in part, for the greater good of healthcare improvement. Bordering on areas of legality and ownership of medical records, however, there have been legitimate concerns of illegal patient data transfer without proper protocol, including informed consent from patients who are deemed as the owners of their own health data in the process of data transfer and exchange [41].

More so, many AI algorithms, particularly deep learning employed for image analysis, are nearly impossible to analyze or explain for the layman. Imagine that a patient is informed that an image has resulted in a cancer diagnosis. Naturally, they would want to understand the cause of such a diagnosis. Deep learning algorithms might be incapable of providing an understandable response [7]. If an AI system makes an error in patient diagnosis and treatment, establishing accountability may be difficult. Hence, the principle of nonmaleficence may be affected. There are chances that patients will obtain medical information from AI systems that they would prefer to receive from an empathetic physician. Finally, despite the advancements that have been made in the field of AI systems and their role in healthcare, it is not readily accessible to everyone, especially to many low-income and developing nations. Since justice in healthcare ethics pertains to the fair distribution of resources and equal access to care, this issue of equitable distribution of AI-driven technologies should be addressed.

The reality of AI in medicine

A plethora of studies have looked into the future trajectory of AI in medicine. According to Ahuja (2019), AI-based technologies will improve diagnostic efficiency, advance robotic surgery, and promote precision medicine in the future [42]. Similarly, Basu et al. (2020) claim that AI systems will improve follow-up visits and the availability of prescription medication alternatives [43].

Notably, AI in healthcare is still at a rudimentary level. However, the truth is that AI has come to stay, and its future in healthcare is promising. Given the influence of AI and machine learning in our society today, it is important that AI be taught to specialists especially in the medical field where making wrong choices have grave consequences [44].

The availability of massive data sets sourced from medical records and wearable health monitors and developments in learning algorithms and computer power will encourage the acceleration of AI in medicine [42]. Powerful algorithms that can integrate diverse structured and unstructured data, such as imaging, electronic health data, multi-omics, and behavioral and pharmaceutical data, may be created in the near future. These algorithms would be efficient (e.g., require less data to train) and powerful. The development of revolutionary AI systems for precision treatments will see healthcare organizations and medical practitioners become co-innovators with technology partners rather than just adopters of AI platforms [28].

Current arguments should steer from how AI has come to replace practitioners to how AI can augment the work of practitioners and enable them to maximize time spent with patients, reduce burnout, and improve patient-centered care. Also, emphasis should be on removing barriers to translating AI in research or laboratory to healthcare and clinical medicine. Just as medical students and residents are now educated to work well with electronic health records, physicians will need to learn how to operate effectively with AI systems so that the integration of AI into healthcare would maximize the quality of care and minimize adverse reactions [44]. A collaborative approach toward AI should be fostered and encouraged because the combination of both human physicians and AI technologies being put to use together can improve healthcare delivery beyond what each entity would have been able to carry out separately [42].

Data is a very important factor to achieving the required potential success expected of AI, but currently, it is unfortunately a major impediment to AI's potential to solve healthcare's most pressing issues [43]. We cannot exploit the full promise of AI in healthcare without sufficient and well-represented data. The need for the digitalization of medical records, the standardization of data infrastructure, the optimization of data quality checks, the promotion of patient confidentiality, and the governance and regulation of the AI life cycle is glaring. In 2018, the American Medical Association (AMA) adopted the Augmented Intelligence in Health Care (H-480.940) policy to ensure the evolution of augmented intelligence [44]. This policy addresses the importance of educating stakeholders on the pros and cons of AI, guarding patients' private information, and exploring the legal significance of AI in healthcare. The policy also calls for the creation of AI systems that comply with the leading standard for reproducibility, are transparent and user-friendly, and can address biases and disparities [44].

Apart from the need for healthcare organizations and countries to develop their policies, achieving a broader international agreement on policies overseeing the implementation of AI in healthcare is paramount. A focus group and semi-structured study conducted by Morley et al. (2022) unveiled four areas in which international agreement would make a difference: leadership and oversight, an ecosystem approach, standards and regulatory processes, and engagement with stakeholders and the public [45]. The consensus among the participants of the study was that there should be an international committee collaborating with national bodies for adequate oversight and to reinforce recommended policies during the AI integration and adoption process [45]. These fundamental changes and interoperability are paramount to a seamless integration of AI into the healthcare industry.

AI will augment rather than replace physicians. AI "cannot engage in high-level conversation or interaction with patients in order to gain their trust, reassure them, or express empathy, all of which are important parts of the doctor-patient relationship" [42]. AI will continue to evolve, and so will the roles of practitioners. AI cannot replace the human element in medicine, but healthcare practitioners must develop competency in AI. One way to achieve this is to formalize AI in the curricula of future practitioners. Based on the results of a multidisciplinary team interview, Russel et al. (2023) described six competency domains that can guide future teachings on AI (Table 1) [26].

**Table 1:** Competency domains AI: artificial intelligence

	Competency domains
1.	Basic knowledge of AI
2.	Social and ethical implications of AI (including social and economic factors that inform AI tools and their effect on equity)
3.	AI-enhanced clinical encounters and how this can optimize patient-centered care
4.	Evidence-based evaluation of AI-based tool
5.	Workflow analysis for AI-based tools
6.	Practice-based learning and improvement regarding AI-based systems

With the rapid progression of AI in healthcare, practitioners who refuse to amass the necessary skill sets may be left behind, while the job market for those who embrace AI will increase.

## Conclusions

This new frontier of machines and humans complementing each other for healthcare advancement has come to stay. Healthcare institutions should strive for a corporate culture for organization-wide adoption. Organizations may benefit from building a team with the expertise to deploy, implement, and use AI in their workflow. Although AI will augment healthcare practitioners and not replace them, it is paramount for healthcare workers to expand their skill sets to keep up with these changing times.

AI systems will revolutionize patient care through accurate diagnosis, precision medicine, and patient-centered care delivery. However, the AI system has to be trained using existing healthcare data to function optimally, thus shedding light on the importance of data availability, sharing, protection, and monitoring. In this review, we highlighted some current and potential areas of application of AI in healthcare, the attitudes of stakeholders, and the misconceptions surrounding the integration of AI into the healthcare industry. Additionally, this review underscores the importance of adequate governance of the AI life cycle.
